# Prevalence and correlates of intimate partner violence among ever-married men in Uganda: a cross-sectional survey

**DOI:** 10.1186/s12889-022-12945-z

**Published:** 2022-03-18

**Authors:** Derrick Gubi, Stephen Ojiambo Wandera

**Affiliations:** 1 Department of Population Studies, School of Statistics and Planning, College of Business and Management Sciences, Makerere University, Kampala, Uganda; 2 Fogarty International Center, National Institutes of Health, Bethesda, Maryland USA

**Keywords:** Intimate Partner violence, Male victims, Violence against men, Uganda

## Abstract

**Background:**

There is limited research on intimate partner violence (IPV) among ever-married men in Uganda. This paper aimed to establish the extent and correlates of emotional, sexual, and physical IPV among ever-married men in Uganda.

**Methods:**

We used the 2016 Uganda Demographic and Health Survey (UDHS) data and selected a weighted sample of 2559 ever-married men. Frequency distributions were used to describe the characteristics of men and their partners. Chi-square tests and binary logistic regressions were used to identify factors associated with IPV among married men in Uganda.

**Results:**

Almost half (44%) of the ever-married men experienced some form of IPV. Among the individual forms of IPV, emotional IPV was the most prevalent (36%), followed by physical IPV (20%) and sexual IPV the least common (8%). Factors that were associated with all the different forms of IPV included, region, number of wives, partners’ controlling behaviors, witnessing parental violence, and drinking alcohol as well as the frequency of getting drunk by the female partners. Except for number of wives, which had a protective effect, the rest of the factors increased the likelihood of experiencing intimate partner violence among ever-married men in Uganda.

**Conclusions:**

Besides women, men are also victims of intimate partner violence. This calls for combined efforts to reduce violence against men perpetrated by females by addressing controlling behaviors, frequency of getting drunk with alcohol, and lack of awareness of the issue. There is a need for interventions aimed at increasing public awareness to improve the reporting and case management of violence against men and boys.

## Background

Intimate partner violence (IPV) is not only a human rights issue but also an economic and public health concern [1, 2]. The World Health Organization (WHO) defines IPV as any behavior within an intimate relationship that causes physical, psychological, or sexual harm to those in the relationship, including acts of physical aggression, sexual coercion, psychological abuse, and controlling behaviors [3]. Also, domestic violence which is at times used interchangeably with IPV, refers to aggressive behavior within a home, usually used to gain or maintain power and control over the partner, and it involves violent abuse of a spouse, ex-partner, and immediate family members like children, parents, and other relatives [4]. IPV remains a big contributor to gender-based violence (GBV) [5].

For a long time, IPV among men has been prevalent but it has not been given the attention it deserves [6, 7]. For instance, Deshpande [8] reported that young men in India with good qualifications and income were often abducted and forced to marry without their consent. In a male-dominated society, like Uganda, men feel that it is shameful to be beaten by a woman and they shun reporting the violence [8, 9]. The probable reasons for under-reporting female perpetrated IPV include social stigma (fear of losing social respect and position), not being believed, fearing shame, having their masculinity questioned, and being accused of domestic violence [10, 11]. Complaining by men is also often perceived as a ‘feminine behavior’, especially in male-dominated societies [8].

The global prevalence of IPV among men is estimated at 17% [12]. Approximately, one in three male victims of IPV sustain serious injuries, lose self-worth, have generally poor health, and resort to alcoholism and drugs [13–15]. In Uganda, the prevalence of IPV among ever-married men is 44% compared to 56% among ever-married women [1]. Some studies like the SASA! community mobilization model in Uganda have compared IPV between men and women, and illustrate pathways of reducing IPV risks [16, 17]. National statistics reveal that violence against men is not only overlooked in Uganda but also underreported, with only half (51%) of the cases being reported [1]. Perhaps the assumption that IPV does not affect men, and the low prevalence of IPV among men could explain the under-investigation of IPV among married men in Uganda.

Furthermore, IPV has been reported among persons with psychosocial/intellectual disabilities in Uganda [1, 18]. For instance, the 2017 Uganda Functional Difficulties Survey (UFDS) reported that physical IPV among male persons with disabilities (PWDs) was 51% and sexual IPV was 25% [19]. These statistics indicate that physical and sexual violence are more prevalent in persons with disabilities than the rest of the population. This could be explained by the marginalization of PWDs, which puts them at increased risk of IPV.

The nested ecological framework theory [20, 21] was used to conceptualize this study. The predictors of IPV among married men are put into context. It looks at the different risk factors for IPV that are intertwined at various levels - individual, relationship, community, and society. At the individual level, the risk factors include alcohol use, witnessing parental violence, and psychological problems [20]. The relationship level factors cover family situation, workplace context, friends, and intimate partners of the married men. In addition, it comprises factors like controlling behaviors of partners, decision making, and marital conflicts [20]. Community level factors (say social status, poverty) highlight the role of the community in which a person lives and develops relations. Society level factors link structures and systems of one’s society to the culture where the individual lives [20, 21]. It constitutes factors like gender roles and norms, acceptance of violence and the linking of masculinity to violence.

Depending on the context, some factors are associated with perpetration, some with victimization, and some with both [6]. Socio-demographics like level of education, wealth status and geographical region have been associated with IPV [12, 18, 22, 23]. History of witnessing parental violence has been found to be a risk factor of IPV [24]. Behavioral factors, on the other hand, especially controlling behaviors of female partners have been linked to IPV, and are said to precede and catalyze IPV [13, 25, 26]. Like marital factors, economic empowerment has been linked to IPV [26]. Economic empowerment was assessed in form of a man owning a house or land (either alone or jointly with a partner) or receiving cash payment for his work.

Most studies carried out in Uganda have been on IPV among women in union [5, 27, 28], and very few have focused on IPV among ever-married men [9]. On a positive note, however, the government of Uganda has put in place policies, laws, and guidelines to provide a legal context within which programs on prevention and response to IPV occur [29]. Such legislative instruments include among others the Domestic Violence Act of 2010 [30], the National Policy on Elimination of Gender-Based Violence [31], and the Uganda Gender Policy of 2007 [32]. The policies underscore IPV as a national concern that impedes development. Despite these efforts, IPV remains persistent in Uganda. In addition to IPV among men being under-investigated in Uganda, men have been silent victims of this violence. Therefore, this study sought to investigate the prevalence and correlates of intimate partner violence among ever-married men in Uganda.

## Methods

### Study design

The paper uses data from the 2016 Uganda Demographic and Health Survey (UDHS), accessed with permission from DHS Program [33]. This was a cross-sectional nationally representative survey that employed a two-stage stratified sampling procedure and cluster sampling design based on the sampling frame from the 2014 National Population and Housing Census [1, 34]. A detailed description of the sampling procedure is reported in the 2016 UDHS report [1].

The sample for the domestic violence (DV) module was 4,032 men. From this sample, we extracted a weighted sample of 2,559 ever-married men (formerly married, currently married or cohabiting), for further analysis. We used the domestic violence weighting variable (dm005) included in the UDHS data and the Stata survey (svy) command to weight the data during the analyses to account for the complex survey design [35].

The domestic violence module was based on the shortened and modified version of the Conflict Tactics Scale (CTS) [36]. The survey was carried out based on the World Health Organization’s (WHO) ethical and safety recommendations for research on domestic violence [37]. Specifically, informed consent was sought from all subjects that took part in the UDHS survey [1] and an institution (International Coaching Federation) approved its survey protocol. The respondents in the UDHS survey were briefed about the purpose of the survey before they could be interviewed. Furthermore, they (respondents) were assured of confidentiality, and their anonymity was ensured.

### Variables and measures

#### Outcome variables

The outcome variables were the three different forms of IPV (physical, sexual, and emotional), which could have different predictors. The paper operationalized physical IPV as any physical act that results in abuse by a current or former partner within 12 months before the survey [27]. Sexual IPV referred to being subjected to sexual abuse, sexual harassment, and/or sexual humiliation by a spouse or former partner within 12 months before the survey [38]. Emotional IPV referred to being systematically and repeatedly degraded, humiliated, insulted, verbally oppressed as well as having one’s dignity violated by a spouse or former partner within 12 months before the survey [38].

In addition, an aggregate measure of IPV (any IPV), which combined all the three forms of violence was generated. Any IPV was operationalized as physical, sexual, or psychological harms as well as controlling behaviors aggravated by a current or former partner within 12 months preceding the survey [5, 39]. Similar outcome variables were used in our earlier publication [5]. The dependent variables were based on the following set of questions asked to men in the survey. Men indicated whether their wives/partners had ever or did;

- Hit, slap, kick or do anything else to hurt them physically?
- Force them to have intercourse or perform any other sexual acts against their will?
- Say something to humiliate them in front of others, threaten to hurt them or someone they care about, insult them or make them feel bad.

The response expected was either ‘yes’ or ‘no’; with ‘yes’ to the questions a, b, and c implying experience of physical, sexual, and emotional IPV respectively and ‘no’ implying non-experience of IPV. In addition, a ‘yes’ to any of the three questions a, b, and c implied experience of any IPV and ‘no’ implied non-experience of any IPV.

### Explanatory variables

In this paper, independent variables were categorized into five categories. First, socio-demographics including men’s age, place of residence, education, region, and wealth status. History of witnessing parental violence was the second category, it was measured by respondents indicating whether their fathers ever beat their mothers, and it had a binary outcome (0 = No, 1 = Yes).

The third group of variables comprised wife/partners’ behaviors, namely, controlling behaviors, alcohol consumption, and frequency of getting drunk. To measure the partner’s controlling behaviors, male participants were asked, “Does your partner ever or did; a) Prohibit you to meet male friends? b) Limit you contact your family? c) Insist on knowing where you are at all times? d) Is jealous if you talk with other women? and e) Frequently accuses you of being unfaithful?” These were merged into one variable called the “partner’s controlling behaviors.” Any affirmative response (yes) to any of the above questions implied the presence of the partner’s controlling behaviors and no to all the questions implied the non-existence of such behaviors. The partner’s alcohol consumption was measured by responses to the question, “Does your partner drink alcohol?” and it had a binary outcome (0 = No, 1 = Yes). The frequency of a partner being drunk was a follow-up question to those respondents whose partners indicated that the partner drank alcohol.

The fourth category of explanatory variables considered marital factors, which included duration of the relationship, number of wives, total children ever born, and age at first marriage. A detailed description of the categorization of these marital variables is reported in our earlier publication [5]. The term “partner” in this paper includes wives as well as partners in cohabiting unions.

The fifth category was on men’s economic empowerment indicators and included their ownership of property (land and house) and type of earning from a man’s work. Ownership of land, as well as a house, was recoded into two categories: man alone/jointly with the partner as the empowered category and partner alone/others as the other. Type of earnings from a man’s work was recoded into binary outcomes - paid either cash only, in-kind only, and cash & in kind as the empowered category and not paid as the non-empowered category. Similar explanatory variables were used in our earlier publication [5] and also in mainland Tanzania [39].

### Statistical analyses

We used frequency distributions to describe the characteristics of respondents. Pearson chi-square tests were used to test initial associations. The level of statistical significance using *p*-values was set at *p*<0.05. Selection of variables was based on the ecological model and published studies about IPV among women, and these variables have been found to be strongly associated with IPV among women [5, 27, 28, 40]. Therefore, we aimed to investigate the possible associations using men’s data. After all, there are few studies about IPV among men.

We tested univariate regressions with variables suggested as strongly associated with IPV. Furthermore, we conducted multivariable logistic regressions to examine the association between selected explanatory variables (with *p*-values less than 0.05) and the experience of emotional, sexual, physical IPV and any form of IPV as an aggregate measure of the three variables. We presented the results in the form of Odds Ratios (OR) and their 95% confidence intervals. All analyses were weighted and performed in Stata version 14.

## Results

### Prevalence of IPV

Among the individual forms of IPV, emotional IPV is the most prevalent (36%), and sexual IPV is the least common (8%). One in 5 men (20%) experienced physical IPV. Overall, almost 5 in 10 (44%) of the men experienced any form of IPV.

### Descriptive characteristics of respondents

Table 1 presents the socio-demographic characteristics of respondents. More than half (78%) of the men resided in rural areas, had primary education (58%), were paid cash or in-kind for their labor, owned a house or land alone or jointly with a partner. There was nearly an even distribution of men by region. The Northern region had the fewest men (20%), while the Eastern region had the highest (28%) representation.

**Table 1:** Socio-demographic characteristics of Respondents

Characteristics	Frequency (n)	Percentage (%)
**Age**		
15-24	297	11.6
25-34	981	38.3
35+	1281	50.1
**Residence**		
Urban	564	22.0
Rural	1995	78.0
**Education**		
No education	145	5.7
Primary	1478	57.7
Secondary+	936	36.6
**Region**		
Central	673	26.3
Eastern	716	28.0
Northern	514	20.0
Western	657	25.7
**Wealth status**		
Poorest	482	18.8
Poorer	496	19.4
Middle	521	20.4
Richer	561	21.9
Richest	499	19.5
**Children ever born**		
None	158	6.0
1-4	1193	47.0
5+	1208	47.0
**Type of earnings from a man’s work**		
Not paid	525	20.5
Paid cash, in-kind or both	2034	79.5
**Ownership of a house alone or jointly**		
Does not own	521	20.4
Owns alone/Jointly	2038	79.6
**Ownership of land alone or jointly**		
Does not own	697	27.3
Owns alone/Jointly	1862	72.8
**Total**	**2559**	**100**

Table 2 presents other IPV-related factors. The majority (77%) of men experienced partner controlling behaviors, married after 18 years of age, married one wife, and had partners that never drank alcohol. Two in 5 (41%) men witnessed parental violence.

**Table 2:** Distribution of IPV related characteristics of the Respondents

Characteristics	Frequency (n)	Percentage (%)
**Partner controlling behaviours**		
No	579	22.6
Yes	1980	77.4
**Partner takes alcohol**		
No	2082	81.4
Yes	477	18.6
**Frequency of partner being drunk**		
Never	2213	86.5
Often	61	2.3
Sometimes	286	11.2
**Witnessing parental violence**		
No	1518	59.3
Yes	1041	40.7
**Duration of relationship**		
0-4 years	623	24.4
5-9 years	526	20.6
10+ years	1410	55.0
**Marital status**		
Married	1543	60.3
Living with partner	795	31.1
Widowed/Divorced/Separated	221	8.6
**Number of wives**		
Formerly married	221	8.7
I wife	2006	78.3
2 or more wives	332	13.0
**Age at first marriage**		
Below 18 years	277	10.8
18+ years	2282	89.2
**Total**	**2559**	**100**

### Association between IPV and explanatory variables

Cross tabulations were conducted to obtain the associations between the different explanatory variables and experience of the different IPV forms. Except for residence, wealth index, age at first marriage, and ownership of land/house, all other variables were significantly associated with either emotional, sexual, physical, or any IPV. Remarkably, monogamous men and those with secondary education or above reported fewer cases of violence. Men whose partners had controlling behaviors and those who witnessed their fathers beat their mothers experienced more IPV. There was an almost even distribution of IPV by wealth index.

### Multivariate results

Table 3 shows the net influence of explanatory variables on the occurrence of emotional, sexual, physical, and any IPV.

**Table 3:** Results of logistic regressions of the different forms of IPV and the explanatory factors

Variables	Model on emotional IPV			Model on sexual IPV			Model on physical IPV			Model on any IPV		
	OR	95% CI	***p***-value	OR	95% CI	***p***-value	OR	95% CI	***p***-value	OR	95% CI	***p***-value
**Age**												
15-24 (RC)	1.00			1.00			1.00			1.00		
25-34	1.03	(0.71-1.50)	0.885	0.86	(0.50-1.46)	0.564	0.97	(0.61-1.55)	0.912	0.94	(0.66-1.35)	0.747
35+	0.99	(0.63-1.56)	0.964	0.63	(0.35-1.15)	0.134	0.83	(0.48-1.42)	0.497	0.98	(0.63-1.53)	0.943
**Education**												
No education (RC)	1.00			1.00			1.00			1.00		
Primary	0.96	(0.61-1.51)	0.861	1.15	(0.51-2.58)	0.733	1.01	(0.62-1.66)	0.958	1.18	(0.75-1.83)	0.475
Secondary+	0.85	(0.51-1.39)	0.513	1.04	(0.44-2.46)	0.922	0.68	(0.40-1.15)	0.148	1.03	(0.63-1.66)	0.912
**Region**												
Central (RC)	1.00			1.00			1.00			1.00		
Eastern	1.14	(0.81-1.59)	0.447	0.72	(0.44-1.19)	0.204	2.08	(1.43-3.02)	**0.000**	1.29	(0.94-1.79)	0.118
Northern	1.29	(0.89-1.87)	0.183	0.42	(0.21-0.87)	**0.019**	1.71	(1.10-2.65)	**0.017**	1.34	(0.92-1.94)	0.127
Western	1.33	(0.97-1.82)	0.075	0.58	(0.37-0.92)	**0.019**	1.67	(1.19-2.34)	**0.003**	1.41	(1.03-1.93)	**0.032**
**Wealth status**												
Poorest (RC)	1.00			1.00			1.00			1.00		
Poorer	1.07	(0.80-1.43)	0.651	1.96	(1.08-3.57)	**0.027**	1.68	(1.16-2.42)	**0.006**	1.23	(0.91-1.65)	0.175
Middle	1.30	(0.92-1.84)	0.132	1.49	(0.80-2.78)	0.204	1.54	(1.02-2.32)	**0.039**	1.37	(0.97-1.93)	0.072
Richer	1.18	(0.83-1.68)	0.346	1.33	(0.69-2.54)	0.391	1.77	(1.15-2.73)	**0.010**	1.30	(0.92-1.83)	0.131
Richest	1.10	(0.72-1.66)	0.664	1.28	(0.59-2.78)	0.539	1.82	(1.10-3.02)	**0.020**	1.19	(0.79-1.79)	0.404
**Type of earning from a man's work**												
Not paid (RC)	1.00			1.00			1.00			1.00		
Paid cash, in-kind or both	1.41	(1.03-1.94)	**0.034**	1.50	(0.87-2.57)	0.144	0.83	(0.60-1.16)	0.275	1.21	(0.89-1.66)	0.230
**Partner takes alcohol**												
No (RC)	1.00			1.00			1.00			1.00		
Yes	1.94	(1.24-3.04)	**0.004**	0.33	(0.13-0.87)	**0.025**	1.26	(0.77-2.07)	0.363	1.62	(1.06-2.49)	**0.027**
**Frequency of partner being drunk**												
Never (RC)	1.00			1.00			1.00			1.00		
Often	2.09	(0.88-5.00)	0.097	6.47	(2.08-20.11)	**0.001**	5.90	(2.84-12.29)	**0.000**	4.47	(1.75-11.41)	**0.002**
Sometimes	1.08	(0.66-1.76)	0.766	3.46	(1.20-9.98)	**0.022**	2.57	(1.48-4.48)	**0.001**	1.42	(0.89-2.26)	0.139
**Witnessing parental violence**												
No (RC)	1.00			1.00			1.00			1.00		
Yes	1.35	(1.09-1.69)	**0.007**	1.26	(0.86-1.85)	0.226	1.20	(0.93-1.54)	0.158	1.42	(1.14-1.76)	**0.001**
**Duration of relationship**												
0-4 years (RC)	1.00			1.00			1.00			1.00		
5-9 years	1.42	(1.04-1.94)	**0.028**	0.85	(0.51-1.42)	0.544	1.19	(0.81-1.74)	0.369	1.17	(0.88-1.55)	0.271
10+ years	1.55	(1.07-2.23)	**0.020**	0.85	(0.53-1.36)	0.492	1.25	(0.84-1.87)	0.261	1.12	(0.81-1.56)	0.481
**Partner’s controlling behaviours**												
No (RC)	1.00			1.00			1.00			1.00		
Yes	6.08	(4.43-8.34)	**0.000**	6.82	(3.73-12.46)	**0.000**	2.75	(1.88-4.02)	**0.000**	4.52	(3.43-5.96)	**0.000**
**Number of wives**												
Formerly married (RC)	1.00			1.00			1.00			1.00		
I Wife	0.44	(0.30-0.65)	**0.000**	0.61	(0.37-1.01)	0.056	0.54	(0.36-0.80)	**0.002**	0.49	(0.34-0.72)	**0.000**
2 or more wives	0.45	(0.29-0.69)	**0.000**	0.61	(0.32-1.18)	0.144	0.59	(0.37-0.94)	**0.028**	0.52	(0.34-0.79)	**0.002**

Bold means *p*-value <0.05

Three socio-demographic variables had a significant relationship with the different forms of intimate partner violence. A man’s geographical region was a significant predictor of sexual, physical, and any IPV. The odds of sexual IPV were lower among men in the Northern (OR=0.42; 95% CI: 0.21-0.87) and Western (OR=0.58; 95% CI: 0.37-0.92) regions of Uganda than in the Central region. The odds of experiencing physical IPV were higher among men in the Eastern (OR=2.08; 95% CI: 1.43-3.02), Northern, (OR=1.71; 95% CI: 1.10-2.65) and Western (OR=1.67; 95% CI: 1.19-2.34) regions of Uganda. Like physical IPV, the odds of experiencing any IPV were higher among men in Western Uganda (OR=1.41; 95% CI: 1.03-1.93).

Wealth status was significantly associated with sexual IPV and physical IPV. Men in the poorer wealth quintile had higher odds (OR=1.96; 95% CI: 1.08-3.57) of experiencing sexual IPV compared to those in the poorest wealth quintile. Also, men in the poorer, middle, richer, and richest wealth quintiles had higher odds (OR=1.68; 95% CI: 1.16-2.42, OR=1.54; 95% CI: 1.02-2.32, OR=1.77; 95% CI: 1.15-2.73 and OR=1.82; 95% CI: 1.10-3.02 respectively) of experiencing physical IPV compared to those in the poorest wealth quintile.

History of witnessing parental violence was associated with emotional IPV and any IPV. Men who witnessed parental violence had higher odds of experiencing emotional IPV (OR=1.35; 95% CI: 1.09-1.69) and any IPV (OR=1.42; 95% CI: 1.14-1.76) compared to those who did not.

All variables in the partners’ behavior category were significant predictors of different forms of IPV. Wife/partners’ controlling behaviors were strongly associated with all forms of IPV. Men who reported partners’ controlling behaviors had higher odds of experiencing emotional IPV (OR=6.08; 95% CI: 4.43-8.34), sexual IPV (OR=6.82; 95% CI: 3.73-12.46), physical IPV, (OR=2.75; 95% CI: 1.88-4.02) and any form of IPV (OR=4.52; 95% CI: 3.43-5.96).

Partner’s alcohol consumption had a significant association with emotional, sexual, and any IPV. Men whose partners drunk alcohol had higher odds of experiencing emotional IPV (OR=1.94; 95% CI: 1.24-3.04) and any IPV (OR=1.62; 95% CI: 1.06-2.49) but lower odds of sexual IPV (OR=0.33; 95% CI: 0.13-0.87).

Related to the above, the frequency of a partner being drunk was a predictor of sexual violence, physical violence, and any IPV. Men whose partners were “often” drunk had higher odds (OR=6.47; 95% CI: 2.08-20.11) of experiencing sexual violence, physical violence (OR=5.90; 95% CI: 2.84-12.29) and any IPV compared to those whose partners were never drunk. Similarly, men whose partners were “sometimes” drunk were more likely (OR=3.46; 95% CI: 1.20-9.98) to experience sexual IPV and physical IPV (OR=2.57; 95% CI: 1.48-4.48).

The marital variables category had two predictors of intimate partner violence, duration of relationship and number of wives. Duration of relationship had a statistically significant relationship with emotional IPV only, with odds of experiencing emotional violence being higher (OR=1.42; 95% CI: 1.04-1.94) among men with 5-9 years’ marital duration and those with 10 or more years’ duration (OR=1.55; 95% CI: 1.07-2.23).

The number of wives a man had was correlated with emotional violence, physical violence, and any IPV. Specifically, men who married one wife were less likely (OR=0.44; 95% CI: 0.30-0.65) to experience emotional IPV, physical IPV (OR=0.54; 95% CI: 0.36-0.80) and any IPV (OR=0.49; 95% CI: 0.34-0.72). Similarly, men with 2 or more wives had lower odds of experiencing emotional IPV (OR=0.45; 95% CI: 0.29-0.69), physical IPV (OR=0.59; 95% CI: 0.37-0.94) and any IPV (OR=0.52; 95% CI: 0.34-0.79).

Among the indicators of economic empowerment, the type of earning from a man’s work was the only predictor of emotional IPV. Men who were either paid cash or in-kind had higher odds (OR=1.41; 95% CI: 1.03-1.94) of experiencing emotional violence compared to those who were not paid.

## Discussion

This study aimed to estimate the prevalence and correlates of IPV among ever-married men in Uganda. The prevalence of IPV among Ugandan men remains relatively high (44%) compared to Kenya [41, 42], India [8], and other developing countries [25, 43, 44]. IPV does not only affect life physically, mentally, emotionally, and psychologically but is also a violation of the basic human rights [8, 39]. A study in India indicated that unreported and unnoticed violence against men often leads to denial in accepting the family, divorce, depression, and at times suicide [8].

We found that female partners’ controlling behaviors are significantly associated with all forms of IPV. Men whose female partners had controlling behaviors had increased odds of experiencing IPV than those whose partners were not. This finding has been reported in previous studies [12, 22] especially from sub-Saharan countries like South Africa [26], Nigeria [25], and developed countries like United Kingdom [13]. Like the context of male-to-female domestic violence, controlling behaviors of females are a precursor to IPV and provide a possibility of violence victimization among men.

Alcohol consumption and the frequency of being drunk by a partner were significant predictors of emotional, sexual, physical, and any IPV. Previous studies among women in Uganda [45–47] reported that alcohol abuse was a precursor to the experience of IPV. Though consumption of alcohol plays a crucial social role in many societies, excessive consumption catalyzes both victimization and perpetration of intimate partner violence.

Socio-demographic factors like men’s wealth status and region of residence were associated with IPV [38]. Men from poor wealth status had increased likelihood of experiencing sexual IPV. In addition, men from all wealth indices had increased odds of experiencing physical IPV. Poverty is associated with strong cultural norms [20] of acceptance of violence. Also, poverty is associated with stress and the pressure to provide for the family / household which lead to reduced interest in sex for some men [46, 48, 49]. Our findings also showed that the odds of experiencing physical IPV were higher among married men from Eastern, Northern, and Western regions of Uganda, compared to those from the Central. Similar findings have been reported in the 2016 Uganda Demographic and Health Survey Report [1, 28, 50]. Also, our findings indicated that men in the Northern and Western regions of Uganda had decreased odds of experiencing sexual IPV compared to those in the Central region. This finding contradicted Schulz [51] who indicated that men and boys suffer sexual violence and torture, especially in humanitarian and crisis environments.

Witnessing of parental violence perpetrated by fathers on the part of respondents was significantly associated with experience of emotional IPV and any IPV. Men who witnessed their fathers beat their mothers were more likely to experience emotional violence as well as any IPV. This finding has been reported in Uganda [52] and elsewhere [43, 53] and is consistent with the social learning theory, which postulates that perpetration and acceptance of violence are conditioned and learned behaviors [20, 54]. Also, such experiences of witnessing parental violence inculcate norms of tolerating or accepting IPV to be part of culture and life. This could explain the concept of intergenerational transmission of IPV [24].

Two of the marital factors were significant predictors of IPV. Specifically, the number of wives was related to emotional, physical, and any IPV. Men who had one or more wives were less likely to experience IPV compared to the widowers who did not remarry. This finding contradicted Kwagala, Wandera [27] as well as Wirtz, Perrin [43] who indicated that having more than one wife divides attention, and at times leads to emotional and economic stress. Perhaps the widely held view among Ugandan men that creation of a family and its stability precedes responsible masculinity [17] could explain this finding. Responsible masculinity, with tenets like marriage, fathering children, providing for family, sexual fidelity, hard work and respect for self and others is defined as conformity to society and large social institutions like family and church [17, 55]. In addition, duration of relationship was significantly associated with emotional IPV, with men having longer durations reporting increased odds of experiencing violence compared to those with shorter durations. Our finding is consistent with the pattern reported in Somalia [43].

One of the economic empowerment indicators - the type of earning from a man’s work, was significantly associated with emotional IPV. Men who were either paid cash or in-kind had increased odds of experiencing emotional violence compared to those who were not paid. Studies in Nigeria [12] and India [56] revealed that less family income was a risk factor of gender-based violence victimization among men.

### Implications

Future studies should focus on one or two sets of explanatory variables while exploring IPV among married men to help gain a deeper understanding of the IPV theory, and why certain factors predict different types of IPV.

Consistent with other patriarchal societies in sub-Saharan countries, we found an association between IPV and a set of factors like alcohol use, witnessing parental violence and controlling behaviors of partners among others. This suggests that interventions to reduce alcohol use and archaic practices of acceptance of violence may be an important pathway to reduce perpetuation of IPV. It may not be possible to eliminate completely the broad social acceptance of violence but it is feasible to formulate regulations and by-laws that regulate alcohol use as well as reforming civil and criminal legal frameworks.

Also, interventions to engage men and boys to promote non-violence, build comprehensive service response to IPV survivors in communities, and organizing media and advocacy campaigns to raise awareness about existing legislations are critical in violence prevention programing.

### Study strengths and limitations

The strength of this study is that it provides a robust estimation of the different forms of IPV among married men using a nationally representative sample. In addition, the paper highlights the predictors of female to male perpetrated IPV – violence against men. However, some limitations are worth mentioning. First, the use of cross-sectional data, where causality among key variables is hard to ascertain is a challenge. Second, self-reporting of different forms of IPV is associated with social desirability biases and underreporting. Third, there is a possibility of potential confounders, say age gap, education gap among others.

## Conclusions

The key predictors of female to male perpetrated IPV included partner’s controlling behaviors, history of witnessing parental violence, number of wives, partner’s alcohol consumption, and frequency of being drunk among others. Indeed, men are also victims of violence in the hands of women. However, their plight is more subtle than that of women. The short and long-term implications of IPV on the health of IPV victims provide a strong case to understand its drivers, to develop prevention programs that target these drivers. Hence, necessary amendments that ensure that male victims of IPV get the same recognition, sympathy, support, and services as their female counterparts should be incorporated by researchers, policymakers, and public health experts. Program interventions that emphasize domestic violence in school curriculum should be developed to respond to and prevent IPV against men as well.

## Data Availability

The datasets generated and/ or analysed during the study are available in the DHS Program repository, available at www.dhsprogram.com.

## Abbreviations

IPV: Intimate Partner Violence
UDHS: Uganda Demographic and Health Survey

## References

[1] Uganda Bureau of Statistics (UBOS) (2018). Uganda Demographic and Health Survey 2016.

[2] Nabukeera, M., The Gender Issues in Uganda: An Analysis of Gender-Based Violence, Asset Ownership and Employment in Uganda. Urban Stud Public Administration, 2020. 3(3).

[3] World Health Organisation (WHO), Violence against women, in Health topics. 2020.

[4] United Nations, An in-depth study on all forms of violence against women. Report of the Secretary General. 2006, United Nations: New York.

[5] Gubi D, Nansubuga E, Wandera SO. Correlates of intimate partner violence among married women in Uganda: a cross-sectional survey. BMC Public Health. 2020;20:1008-18. 10.1186/s12889-020-09123-4 PMC7318470 32586297

[6] World Health Organisation, Understanding and addressing violence against women. 2012.

[7] Hogan, K., Men’s experiences of female-perpetrated intimate partner violence: A qualitative exploration, in School of Psychology, Faculty of Health and Social Sciences. 2016, University of the West of England.

[8] Deshpande S (2019). Sociocultural and Legal Aspects of Violence Against Men. J Psychosexual Health.

[9] Kyamulabi A (2021). ’Turning the Tables’ in COVID-19: Violence against Men in Kampala Slums.

[10] Cheung M, Leung P, Tsui V (2009). Asian Male Domestic Violence Victims: Services Exclusive for Men. J Fam Violence.

[11] James T. Domestic violence against men is the most under reported crime. 2012.

[12] Lanre AO, et al. Assessment of prevalence and forms of violence against married men in Olorunda Local Government of Osun State, Nigeria. Int J Soc Behav Sci. 2014;2(1):001-010.

[13] Hine B, Bates EA, Wallace S. “I Have Guys Call Me and Say ‘I Can’t Be the Victim of Domestic Abuse’”: Exploring the Experiences of Telephone Support Providers for Male Victims of Domestic Violence and Abuse. J Interpersonal Violence. 2020. p. 1-32. 10.1177/0886260520944551. 10.1177/0886260520944551 PMC8980445 32727270

[14] Hines DA, Douglas EM (2015). Health problems of partner violence victims: Comparing help-seeking men to a population-based sample. Am J Prevent Med.

[15] Hines DA, Douglas EM (2016). Relative influence of various forms of partner violence on the health of male victims: Study of a help seeking sample. Psychol Men Masculinity.

[16] Abramsky T, et al. Ecological pathways to prevention: How does the SASA! community mobilisation model work to prevent physical intimate violence against women? BMC Public Health. 2016;16:339-59. 10.1186/s12889-016-3018-9 PMC4833941 27084116

[17] EunheePark et al. Examining Masculinities to Inform Gender-Transformative Violence Prevention Programs: Qualitative Findings From Rakai, Uganda. Global Health: Sci Pract. 2022;10(1):1-12. 10.9745/GHSP-D-21-00137 PMC8885339 35044929

[18] Tanabe M (2015). Intersecting Sexual and Reproductive Health and Disability in Humanitarian Settings: Risks, Needs, and Capacities of Refugees with Disabilities in Kenya, Nepal, and Uganda. Sex Disabil.

[19] Uganda Bureau of Statistics (2018). Uganda Functional Difficulties Survey 2017.

[20] Ali, P.A. and P.B. Naylor, Intimate partner violence: A narrative review of the feminist, social and ecological explanations for its causation. Elsevier, 2013: p. 611–619.

[21] Shorey, R.C., T.L. Cornelius, and K.M. Bell, A critical review of theoretical frameworks for dating violence: Comparing the dating and marital fields. Elsevier, 2008.

[22] Dolan C (2014). Into the mainstream: addressing sexual violence against men and boys in conflict.

[23] Hershow RB (2020). Perpetration of Intimate Partner Violence Among Men Living with HIV in Northern Vietnam. AIDS Behavior.

[24] Hindin, M., S. Kishor, and D.L. Ansara, Intimate partner violence among couples in 10 DHS countries: predictors and health outcomes. DHS Analytical Studies 18, 2008.

[25] Dienye PO, Gbeneol PK (2009). Domestic Violence Against Men in Primary Care in Nigeria. Am J Men’s Health.

[26] Victor, L.N. and N.V. Olive, An investigation into the trend of Domestic Violence on Men: Re-visiting the Sepedi Adage “Monna Ke Nku O llela Teng”. 2019.

[27] Kwagala B, et al. Empowerment, partner’s behaviours and intimate partner violence among married women in Uganda. BMC Public Health, 2013;13:1112-22. 10.1186/1471-2458-13-1112 PMC4219526 24289495

[28] Wandera, S., et al., Partners’ controlling behaviors and intimate partner sexual violence among married women in Uganda. BioMed Central, 2015. 10.1186/s12889-015-1564-1 PMC4350635 25884572

[29] Ministry of Gender Labour and Social Development (2017). The national male involvement strategy for the prevention and response to gender based violence in Uganda.

[30] Uganda, R.o., The Domestic Violence Act 2010. 2010.

[31] Ministry of Gender Labour and Social Gevelopment, The National Policy on Elimination of Gender Based Violence in Uganda. 2016: Kampala.

[32] Uganda, G.o., The Uganda Gender Policy (2007). 2007.

[33] Measure DHS, Demographic and Health Surveys. 2018.

[34] Uganda Bureau of Statistics (UBOS) (2016). The National Population and Housing Census 2014- Main report.

[35] StataCorp (2014). Stata statistical software: Release 13.

[36] Straus, M.A., et al., The revised Conflict Tactics Scales (CTS). Development and preliminary psychometric data. 1996.

[37] World Health Organisation (WHO), Putting women first: Ethical and safety recommendations for research on domestic violence against women. 2001: Geneva.

[38] Ahnlund P, et al. Prevalence and Correlates of Sexual, Physical, and Psychological Violence Against Women and Men of 60 to 74 Years in Sweden. J Interpersonal Violence. 2017;1(23):1-23. 10.1177/0886260517696874 29294678

[39] Kazaura MR, Mangi JE, Chitama D. Magnitude and factors associated with intimate partner violence in mainland Tanzania. BMC Public Health. 2016;16:497-503. 10.1186/s12889-016-3161-3 PMC4902958 27286859

[40] Kwagala B, et al. Empowerment, intimate partner violence and skilled birth attendance among women in rural Uganda. BioMed Central. 2016;13:53-61. 10.1186/s12978-016-0167-3 PMC4855711 27141984

[41] Haushofer, J., et al. Spousal Disagreement in Reporting of Intimate Partner Violence in Kenya. in AEA Papers and Proceedings 2020. 2020.

[42] National Gender and Equality Commission, Gender-based violence in Kenya: The economic burden on survivors. 2016.

[43] Wirtz AL, et al. Lifetime prevalence, correlates and health consequences of gender-based violence victimisation and perpetration among men and women in Somalia. BMJ Global Health, 2018;3:1-12. 10.1136/bmjgh-2018-000773 PMC6074632 30105094

[44] Chynoweth, S., Sexual violence against men and boys in the Syria crisis. 2017, United Nations High Commissioner for Refugees (UNHCR).

[45] Tumwesigye N, et al. Problem drinking and physical intimate partner violence against women: evidence from a national survey in Uganda. BMC Public Health. 2012;12:399-409. 10.1186/1471-2458-12-399 PMC3406983 22672439

[46] Wandera B, et al. Alcohol consumption among HIV Infected persons in a large urban HIV clinic in Kampala Uganda: A constellation of harmful behaviors. PLoS One. 2015;10(5):1-16. 10.1371/journal.pone.0126236 PMC4427399 25962171

[47] Black E, et al. Prevalence and correlates of intimate partner violence against women in conflict affected northern Uganda: a cross-sectional study. Conflict Health. 2019;13:35-44. 10.1186/s13031-019-0219-8 PMC6668065 31384294

[48] Gibbs A, et al. Associations between poverty, mental health and substance use, gender power, and intimate partner violence amongst young (18-30) women and men in urban informal settlements in South Africa: A cross-sectional study and structural equation model. PLOS ONE. 2018;13(10):1-19. 10.1371/journal.pone.0204956 PMC6169941 30281677

[49] Tumwesigye N, Kasirye R, Nansubuga E. Is social interaction associated with alcohol consumption in Uganda? Drug and Alcohol Dependence. 2009;103(1):9-14. 10.1016/j.drugalcdep.2009.01.016 19406589

[50] Wandera, S.O., J.P.M. Ntozi, and B. Kwagala, Spousal sexual violence, sexual behavior and sexually transmitted infections among ever-married women in Uganda. Afr Popul Stud 2010. 24(1 & 2).

[51] Schulz P (2018). The “ethical loneliness” of male sexual violence survivors in Northern Uganda: gendered reflections on silencing. Int Feminist J Politics.

[52] Speizer IS (2010). Intimate partner violence attitudes and experience among women and men in Uganda. J Interpers Violence.

[53] Samuels F (2017). Men and intimate partner violence: From research to action in Bangladesh, Nepal and Pakistan.

[54] Bandura A (1973). Aggression: a social learning analysis..

[55] Wilson PJ (1969). Reputation and respectability: a suggestion for Caribbean ethnology. Man.

[56] Malik JS, Nadda A. A Cross-sectional Study of Gender-Based Violence against Men in the Rural Area of Haryana, India. Indian J Community Med. 2019;44:35-8. 10.4103/ijcm.IJCM_222_18 PMC6437789 30983711

